# Genomic Insights into *Marinovum sedimenti* sp. nov., Isolated from Okhotsk Sea Bottom Sediments, Suggest Plasmid-Mediated Strain-Specific Motility

**DOI:** 10.3390/microorganisms14010125

**Published:** 2026-01-07

**Authors:** Lyudmila Romanenko, Viacheslav Eremeev, Evgeniya Bystritskaya, Peter Velansky, Valeriya Kurilenko, Marina Isaeva

**Affiliations:** 1 G.B. Elyakov Pacific Institute of Bioorganic Chemistry, Far Eastern Branch, Russian Academy of Sciences, Prospect 100 Let Vladivostoku, 159, Vladivostok 690022, Russia; wieremeew@gmail.com (V.E.); ep.bystritskaya@yandex.ru (E.B.); valerie@piboc.dvo.ru (V.K.); 2 A.V. Zhirmunsky National Scientific Center of Marine Biology, Far Eastern Branch, Russian Academy of Sciences, Palchevskogo Street 17, Vladivostok 690041, Russia; velansky.pv@gmail.com

**Keywords:** *Marinovum sedimenti* sp. nov., *Roseobacteraceae*, marine bacteria, sediments, chromosome, plasmids, pan-genome analysis

## Abstract

Two Gram-negative aerobic halophilic bacteria, designated KMM 9989^T^ and KMM 9879, were isolated from a bottom sediment sample of the Okhotsk Sea, Russia. The novel strains grew in 0.5–4% NaCl, at 5–35 °C and pH 5.5–10.0. Phylogenetic analyses based on 16S rRNA gene and whole genome sequences placed strains KMM 9989^T^ and KMM 9879 within the family *Roseobacteraceae*, where they were clustered with their closest relative *Marinovum algicola* KCTC 22095^T^. The average nucleotide identity (ANI) between strain KMM 9989^T^ and *Marinovum algicola* KCTC 22095^T^ was 81.4%. The level of digital DNA–DNA hybridization (dDDH) between the novel isolates KMM 9989^T^ and KMM 9879 was 97%, while between strain KMM 9989^T^ and *Marinovum algicola* KCTC 22095^T^, it was 27%. Strains KMM 9989^T^ and KMM 9879 contained Q-10 as the predominant ubiquinone and C_18:1_*ω7c* as the major fatty acid. The polar lipids were phosphatidylcholine, phosphatidylglycerol, phosphatidylethanolamine, diphosphatidylglycerol, an unidentified aminolipid, two unidentified phospholipids, and three unidentified lipids. The genomic size of strains KMM 9989^T^ and KMM 9879 was determined to be 4,040,543 bp and 3,969,839 bp with a DNA GC content of 61.3 and 61.4 mol%, respectively. Both strains contained a common plasmid of 238,277 bp and a strain-specific plasmid (188,734 bp for KMM 9989^T^ and 118,029 bp for KMM 9879). It is suggested that the motility of KMM 9879 may be mediated by the presence of a complete *fla2*-type operon in the strain-specific chromid. Thus, based on the phylogenetic analyses and distinctive phenotypic characteristics, the novel marine strains KMM 9989^T^ and KMM 9879 are proposed to be classified as a novel species *Marinovum sedimenti* sp. nov. with the strain KMM 9989^T^ (=KCTC 8835^T^) as the type strain of the species.

## 1. Introduction

The genus *Marinovum* was described by [1] as a result of the reclassification of *Ruegeria algicola* [2] previously classified by [3] as *Roseobacter algicola*. At the time of writing, the genus *Marinovum* comprised only one species *Marinovum algicola* FF3^T^, isolated from the phycosphere of the toxin-producing dinoflagellate *Prorocentrum lima*. One more species, “*Marinovum faecis*,” was proposed for the strain YP194^T^ isolated from a marine sediment sample collected at Deokdong Beach, Yokji Island, Republic of Korea, but this scientific name has not yet been validated [4].

The genus *Marinovum* belongs to the family *Roseobacteraceae*, which was proposed by Liang et al. (2021) [5] based on the combined whole-genome phylogenetic and genotypic analyses of the so-called “Roseobacter” bacterial group of the *Rhodobacteraceae* family [6]. These bacteria have been reported to be widespread microorganisms in marine environments, being recovered from seawater, sediments, polar sea ice, seaweeds, and hydrobionts [7,8]. Based on metagenomic analysis, it was established that *M. algicola* and its relatives are highly abundant and are found in oceanic plankton samples [9]. The high-quality circular genome assemblies of *M. algicola* strains have shown that they contain an unprecedented number and diversity of extrachromosomal replicons (ECRs), with 28.5% of genes located on chromids and plasmids [10,11]. These multipartite genomes provide extraordinary metabolic potential and adaptation to the phycosphere [11]. Flagellar motility in *Rhodobacterales* is essential for their “swim-and-stick” lifestyle, facilitating bacterial-algal interactions. The flagellar system can be encoded by three distinct flagellar gene clusters (FGCs): *fla1*, *fla2*, and *fla3.* It was shown that the *fla1*-type FGC is archetypal and vertically inherited, while the *fla2* and *fla3* FGCs are subject to horizontal gene transfer [12]. Specifically, the multipartite genome of *M. algicola* DG898, another strain isolated from the toxic dinoflagellate *Gymnodinium catenatum* YC499B15, demonstrates the presence of both chromosome-encoded (*fla1*-type FGC) and chromid-encoded (*fla2*-type FGC) flagella, which is indispensable for swimming motility [11].

In this study, we report the isolation, phenotypic, and phylogenomic characterization of two novel strains, designated KMM 9989^T^ and KMM 9879, which were found during the cultivation of bacteria dwelling in the bottom sediments of the Okhotsk Sea. Phylogenetic analyses based on the 16S rRNA gene and whole genome sequences demonstrated that the novel isolates were closely related to each other, and their closest relative was detected to be *M. algicola* within the family *Roseobacteraceae*. Strains KMM 9989^T^ and KMM 9879 were spherical or ovoid-shaped bacterial cells, while KMM 9989^T^ was non-motile, and KMM 9879 was motile with one to two polar or lateral flagella.

Based on combined phylogenomic analysis and distinctive phenotypic characteristics, a novel species is described to accommodate strains KMM 9989^T^ and KMM 9879, for which the name *Marinovum sedimenti* sp. nov. is proposed. The type strain of the species is strain KMM 9989^T^.

## 2. Materials and Methods

### 2.1. Bacterial Strains

Strains KMM 9989^T^ and KMM 9879 were isolated from a bottom sediment sample by a standard dilution plating method on marine agar 2216 (MA; BD Difco) at 28 °C. Sampling was carried out at a depth of 31.9 m (54.187933, 137.831566), seawater temperature of 10.1 °C, and salinity of 30.2 ppm in the Okhotsk Sea, Russia, during the expedition of the R/V Academician Oparin in September 2020. The novel bacteria were cultivated aerobically on marine agar 2216 (MA; BD Difco^TM^, Sparks, MD, USA) or in marine broth (MB; BD Difco^TM^, Sparks, MD, USA) 2216 (BD Difco) at 28 °C and stored at −70 °C in MB 2216 supplemented with 20% (*v/v*) glycerol. Strains KMM 9989^T^ and KMM 9879 have been deposited in the Collection of Marine Microorganisms (KMM), Russia, and strain KMM 9989^T^ was deposited in the Korean Collection for Type Cultures (KCTC), Korea, under the number KCTC 8835^T^. The type strain of *M. algicola* KCTC 22095^T^ was purchased from the Korean Collection for Type Cultures to be applied in the comparative phenotypic analyses.

### 2.2. Phenotypic Characterization

Gram-staining, oxidase and catalase reactions, and motility (the hanging drop method) were determined as described by [13]. An oxidase reagent kit (bioMérieux, Marcy-l’Étoile, France) was used for testing oxidase activity according to the manufacturer’s instruction. To analyze cell morphology, strains were grown in MB 2216 for 24 h at 28 °C; the cells were in the logarithmic growth phase. The cell morphology and flagella presence were examined using a transmission electron microscope as described by [14] with 1% phosphotungstic acid on carbon-coated 200-mesh copper grids using a Libra 120 FE (Carl Zeiss, Oberkochen, Germany), provided by A. V. Zhirmunsky National Scientific Centre of Marine Biology, FEB RAS. Hydrolysis of starch, casein, gelatin, Tweens 20, 40, and 80, and L-tyrosine; nitrate reduction (sulfanilic acid/α-naphthylamine test); formation of H_2_S from thiosulfate; and growth at different salinities (0–12% NaCl), temperatures (4–40 °C), and pH values (4.5–10.5) were tested using artificial sea water (ASW) as described in a previous paper [15]. The ASW contained the following (per liter of distilled water): 24 g NaCl, 4.9 g MgCl_2_, 2.0 g MgSO_4_, 0.5 g CaCl_2_, 1.0 g KCl, 0.01 g FeSO_4_. Hydrolysis of DNA was examined using DNase Test Agar (BD BBL^TM^, Sparks, MD, USA). The formation of a transparent zone around a spot of bacterial cells was considered a positive result. Biochemical tests were performed at pH 7.0–7.5 unless stated otherwise using API 20E, API ID32 GN, and API ZYM test kits (bioMérieux, Marcy-l’Étoile, France) as described by the manufacturer, except the cultures were suspended in ASW and are listed in the species description in details.

### 2.3. Chemotaxonomic Analyses

Strains KMM 9989^T^, KMM 9879, and *M. algicola* KCTC 22095^T^ were grown on MA 2216 at 28 °C for 3 days. Lipids were extracted as described by [16], and polar lipids were analyzed using a two-dimensional thin-layer chromatograph according to [17,18]. Fatty acid methyl esters were obtained according to the MIDI method [19] and detected on a GC-2010 chromatograph (Shimadzu Corporation, Kyoto, Japan) with a flame ionization detector and a GC-MS QP2020 (Shimadzu Corporation, Kyoto, Japan) as described in a previous work [20]. Identification of double-bond and methyl group positions in fatty acids was determined according to [21]. Isolation and analysis of quinones were carried out by HPLC as described by [22] on a Shimadzu LC-30 chromatograph (Shimadzu Corporation, Kyoto, Japan) with a photodiode array detector SPD-M30A (Shimadzu Corporation, Kyoto, Japan). Production of bacteriochlorophyll *a* (Bchl *a*) was spectrophotometrically tested in methanolic extracts of cells grown on MA and MB in the dark as described by Lafay et al. (1995) [3].

### 2.4. 16S rRNA Gene Sequence and Phylogenetic Analysis

The extraction of genomic DNA from strains KMM 9989^T^ and KMM 9879 was performed using the NucleoSpin Tissue kit (Macherey–Nagel, Düren, Germany), and the 16S rRNA gene was amplified and sequenced as described in [23]. The 16S rRNA gene sequences were used for initial strain identification utilizing the EzBioCloud service, accessed on 13 May 2025 [24]. Phylogenies were computed on the GGDC web server (https://ggdc.dsmz.de/phylogeny-service.php, accessed on 10 September 2025) [25] using the DSMZ phylogenomics pipeline [26] adapted to a single gene. The inference of maximum likelihood (ML) and maximum parsimony (MP) trees was conducted from RAxML version 8.2.12 [27] and TNT [28] alignments, respectively. The reliability of the trees was assessed using the bootstrap of 1000 replicates.

### 2.5. Whole-Genome Sequencing, Phylogenomic, and Comparative Analyses

Genomic DNA from strains KMM 9989^T^ and KMM 9879 was obtained with the Monarch^®^ Genomic DNA Extraction Kit (New England Biolabs, Ipswich, MA, USA). The DNA sequencing libraries were prepared with a Nextera DNA Flex kit (Illumina, San Diego, CA, USA). Sequencing was performed utilizing paired-end reads on an Illumina MiSeq platform with a 150 bp read length. The libraries for nanopore genome sequencing were obtained with a SQK-NBD114.96 kit (Oxford Nanopore Technologies, Oxford, UK) and sequenced on the MinION, flow cell FLO-MIN 114 (Oxford Nanopore Technologies, Oxford, UK). Basecalling was performed using Dorado (v. 1.0.2). Resulting short and long reads were trimmed and filtered using Trimmomatic (quality over 30, length over 100) version 0.39 [29] with the following parameters: PE, HEADCROP:15, SLIDINGWINDOW:4:30, MINLEN:100, AVGQUAL:30. Long reads were processed using chopper (quality over 16, length over 2000) version 0.10.0 [30] with the following settings: -q 16, --minlength 2000. The quality of processed reads was assessed with FastQC version 0.11.8 (https://www.bioinformatics.babraham.ac.uk/projects/fastqc/, accessed on 21 August 2021 and 30 November 2023). The filtered reads were used for hybrid assembly with Autocycler version 0.5.0 [31]. The pipeline facilitated the obtainment of four subsets of long reads, which were then assembled independently using Flye version 2.9.2 [32], Canu version 2.2 [33], miniasm version 0.3 [34], NextDenovo version 2.5.1 [35], plassempler version 1.8.0 [36] and raven version 1.8.3 [37] as advised by the Autocycler manual. Additionally, the same four subsets underwent a hybrid assembly with a set of short reads using unicycler version 0.5.0 [38] with default parameters. All of the resulting assemblies were then combined into a single consensus assembly, which was additionally polished with pilon version 1.24 [39]. Sequencing depth was estimated utilizing SAMtools version 1.3 [40]. Genome completeness and contamination were estimated by CheckM version 1.1.3 based on the taxonomic-specific workflow [41].

The Average Nucleotide Identity (ANI), Average Amino Acid Identity (AAI), and digital DNA-DNA hybridization (dDDH) values between KMM 9989^T^, KMM 9879 and their closest neighbors were estimated by the fastANI [42], EzAAI version 1.2.4 [43], and TYGS platforms [44], respectively. The phylogenomic analysis was conducted with PhyloPhlAn software version 3.0.1 based on a set of 400 conserved bacterial protein sequences [45]. The selection of bacterial genomes was based on the results of AAI-profiler [46], TYGS [44], and DSMZ phylogenetic pipeline [26]. The list of selected bacterial genomes is given in Table S1.

The annotation of genomes was carried out by the NCBI Prokaryotic Genome Annotation Pipeline (PGAP) [47], Rapid Annotation using Subsystem Technology (RAST) [48], and Prokka version 1.14.6 [49]. To visualize the circular genome of the KMM 9989^T^ and KMM 9879 strains, the Proksee platform was used [50]. Putative Horizontal Gene Transfer (HGT) events were detected via Alien Hunter [51]. CRISPR arrays and associated Cas proteins were found using CRISPR/Cas Finder version 4.2.20 [52]. Replication origin was predicted by Ori-Finder 2022 [53].

Identification of the secretion system (T1SS, T2SS, TAD) components was carried out by MacSyFinder version 2.1.6 (TXSScan models) [54]. Prediction of carbohydrate-active enzymes (CAZymes) was performed using a dbCAN3 meta server with the default settings (https://bcb.unl.edu/dbCAN2/index.php, accessed on 14 November 2025) [55].

Pan-genomic analysis and metabolism estimation were performed by anvi’o version 8 [56]. The genome sequences, both obtained from NCBI and acquired during this study, were reformatted and imported into anvi’o as contig-dbs using internal scripts. The annotation of contigs-dbs inside the anvi’o environment was carried out via the “anvi-run-kegg-kofams” command using the snapshot of the KEGG database from 22 September 2023 [57]. Consequently, an “anvi-estimate-metabolism” command was run with the “--include-metadata” and “--matrix-format” flags. The resulting tables were analyzed manually using Microsoft Excel and compared with KAAS annotation data [58]. The pan-genome was reconstructed using the workflow described at https://merenlab.org/2016/11/08/pangenomics-v2/, accessed on 18 November 2025. PanGP version 1.0.1 was used to visualize cumulative curves [59]. The pan-genome openness was estimated under Heap’s law model [60]. Fonts and sizes in all figures were edited manually in Adobe Photoshop CC 2018 for better visualization.

Codon usage for KMM 9989 and KMM 9879 replicons was calculated using an online calculator available at https://www.bioinformatics.org/sms2/codon_usage.html, accessed on 18 December 2025 [61]. The gene FGCs were visualized with clinker version 0.0.31 [62].

## 3. Results

### 3.1. Phylogenetic and Phylogenomic Analysis

The analysis of 16S rRNA gene sequence similarity, performed by the EzBioCloud service [21], revealed the strains KMM 9989^T^ (1344 bp, PP217372) and KMM 9879 (1271 bp, PP217369) to be close to *Seohaeicola saemankumensis* SD-15^T^ (97.4%), *Pseudophaeobacter flagellatus* MA21411-1^T^ (97.3%), *Phaeobacter porticola* P97^T^ (97.2%), *Phaeobacter inhibens* DSM 16374^T^ (97.0%), and *Phaeobacter gallaeciensis* DSM 26640^T^ (97.0%). The other members of the family *Roseobacteraceae* shared less than 96.9% similarity.

**Figure 1:**
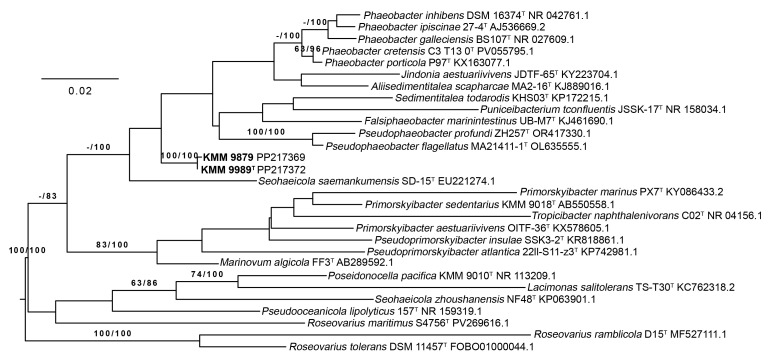
ML/MP tree based on 16S rRNA gene sequences available from the GenBank database showing relationships of the novel strains KMM 9989^T^ and KMM 9879 (in bold), *Marinovum* species, and related taxa of the family *Roseobacteraceae*. The branches are scaled in terms of the expected number of substitutions per site. The numbers above the branches represent bootstrap values with 1000 replicates larger than 60% (ML/MP). The bar indicates 0.02 accumulated substitutions per nucleotide position.

**Figure 2:**
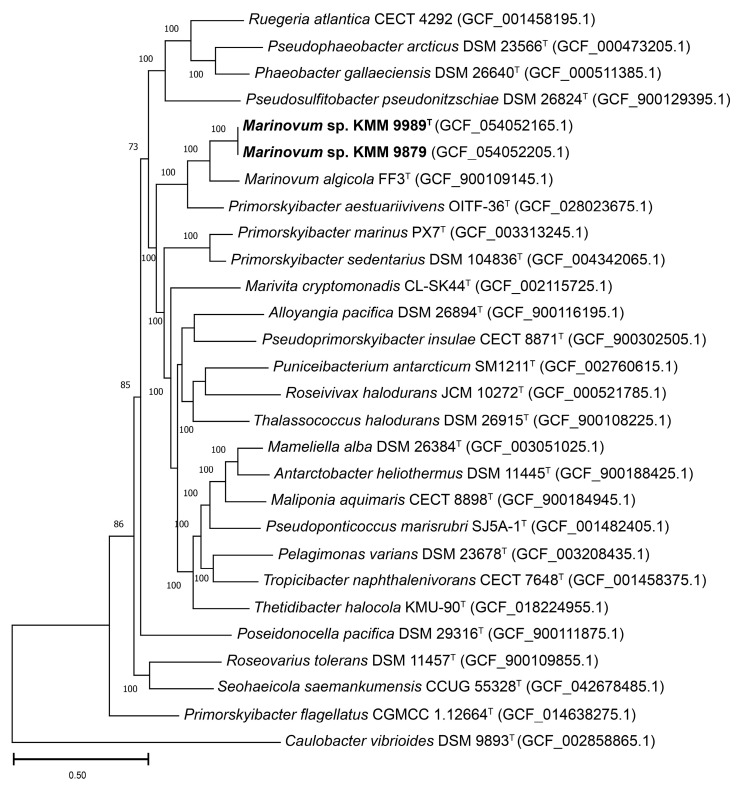
ML tree based on concatenated sequences of 400 proteins showing phylogenetic position of strains KMM 9989^T^ and KMM 9879 (shown in bold) among *Roseobacteraceae*-related taxa. Bootstrap values are based on 100 replicates. Bar, 0.50 substitutions per amino acid position.

**Figure 3:**
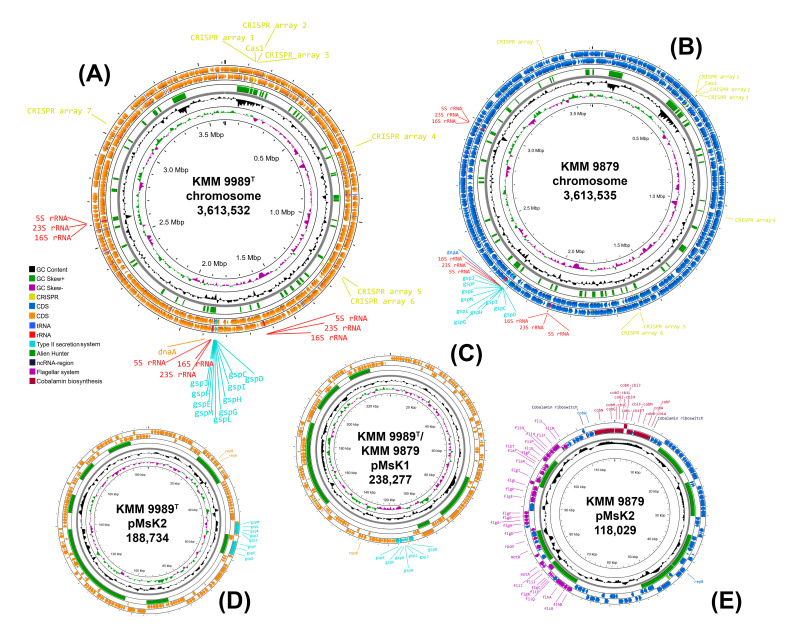
Circular replicon maps of KMM 9989^T^ and KMM 9879 created using the Proksee server [50]. (**A**,**B**) chromosome maps; (**C**) common plasmid pMsK1; (**D**,**E**) strain-specific plasmid pMsK2. Starting with the inner rings, the first two circles represent GC content (in black) and GC skew (G − C)/(G + C) (in violet blue and light green). The middle circle (in green) represents Alien Hunter hits. The last two circles show reverse and forward strand CDSs, as well as *rrn* operons (red labels), CRISPR arrays and CRISPR-Cas regions (yellow labels), replication initiators (orange labels), *gsp* loci or T2SS operons (turquoise labels), *fla*2-type FGC (violet labels), and cobalamin biosynthesis genes (dark red labels).

Phylogenetic trees of the 16S rRNA gene sequences were constructed using the TYGS platform [41]. According to these trees, KMM 9989^T^ and KMM 9879 formed a separated branch, adjacent to the branches of the already-mentioned type strains *S. saemankumensis* SD-15^T^, *Phaeobacter* spp., and *Pseudophaeobacter* spp. (Figure 1). However, their position was inconclusive due to low ML/MP bootstrap supports (less than 60%). In the list of the top 50 hits from the EzBioCloud 16S rRNA database, no representatives of the genera *Primorskyibacter* and *Pseudooceanicola* were found, which are represented on the trees. It should be noted that the novel strains KMM 9989^T^ and KMM 9879 showed 96.4% sequence similarity to the type strain *Marinovum algicola* DSM 10251^T^, which in further phylogenomic analysis turned out to be the closest relative of these strains (Figure 2). Although the 16S rRNA gene sequence coverage was 91.4% for KMM 9989^T^ and 86% for KMM 9879, the full-length 1471 bp 16S rRNA gene sequences showed the same similarity values with all the above-mentioned type strains. This finding suggests that in the case of the new strains, similarity at the 16S rRNA gene level does not reflect relatedness at the genomic level, possibly because all three *rrn* operon copies were located in regions of putative HGT events (Figure 3).

To accurately define the phylogenetic position of the new strains KMM 9989^T^ and KMM 9879, a phylogenomic tree was constructed based on concatenated sequences of 400 proteins retrieved from corresponding genomes (Figure 2). The choice of genomes was guided by AAI-profiler [46] and GBDP tree (whole-proteome-based) [44] results (Figure S1), as well as 16S rRNA strain typing (Figure 1). The resulting phylogenomic tree revealed that strains KMM 9989^T^ and KMM 9879 form a distinct branch neighboring the type strains *M. algicola* FF3^T^ and *Primorskyibacter aestuariivivens* OITF-36^T^ [63]. It is also worth noting that *P. aestuariivivens* did not cluster with the type species of the genus *Primorskyibacter*, *P. sedentarius* DSM 104836^T^. Remarkably, KMM 9989^T^ and KMM 9879 strains did not group on the 16S rRNA gene phylogenetic tree with the members of *Marinovum* and *Primorskyibacter* genera (Figure 1).

The ANI/AAI values between strains KMM 9989^T^ and KMM 9879 were almost 100%, while the dDDH value was 97.1% (formula d0) or 100% (formula d4). The overall genomic relatedness indices (OGRIs) values between KMM 9989^T^ and type strains *M. algicola* KCTC 22095^T^ and *P. aestuariivivens* OITF-36^T^ were 81.4% and 79.2% for ANI, 81.1% and 76.7% for AAI, and 27.1% and 18.5 for dDDH (formula d0), respectively.

It is worth noting that determination of the phylogenetic position of *Primorskyibacter flagellatus* [5,64,65] was hindered by inconsistencies in the genomic data provided for the two type strains, CGMCC 1.12644^T^ and CGMCC 1.12664^T^. The former appears to be *P. flagellatus* (93.5% ANI similarity with *Primorskyibacter marinus* PX7^T^), while the latter is supposed to be *Pseudooceanicola flagellatus* (82–85% ANI similarity with the *Pseudooceanicola* members). To address this discrepancy, the additional phylogenomic tree was constructed on a small set of type strains of *Primorskyibacter*, *Marinovum*, and *Pseudooceanicola* (Figure S2).

Thus, based on the phylogenomic position of strains KMM 9989^T^ and KMM 9879 together with the obtained OGRIs, it was suggested that these novel strains represent a novel species in the genus *Marinovum*.

### 3.2. Genomic Characteristics and Pan-Genome Analysis

The genomes of both strains KMM 9989^T^ and KMM 9879 were assembled de novo into complete closed ones with one chromosome and two plasmids each. The chromosome and plasmid maps were obtained and visualized through Proksee [50] (Figure 3). The genomic sizes of KMM 9989^T^ and KMM 9879 were 4,040,543 bp and 3,969,839 bp with an overall GC content of 61.3% and 61.4%, respectively. Each genome contained three *rrn* operons (gene order is 16S-23S-5S). For both strains, the 16S rRNA gene copies obtained from the genome data differed by a single mismatch between themselves, which corresponded to the PCR-amplified fragments (PP217372). When comparing the genomic sequences, a high level of conservation was observed across the entire chromosome and the first common plasmid (238,237 bp), while the second plasmid differed significantly between strains in both size (188,734 bp for KMM 9989^T^ and 118,029 bp for KMM 9879) and gene content (Figure 3, Table 1).

For comparative genomic analysis, the genome sequences of six strains were used (Table 1), including three strains of *M. algicola* (FF3^T^, DG 1292, and DG 898) and type strain *P. aestuariivivens* OITF-36^T^, which occupied a neighboring branch to the *Marinovum* clade and had more similar OGRIs to them. The genome size of the new strains was about 4 Mb, which is significantly less than those of *M. algicola* strains and slightly less than that of *P. aestuariivivens* OITF-36^T^. The differences were observed in the number of *rrn* operons between the new species and *M. algicola*, as well as in the number of ECRs. Based on the comparison of complete genome sequences, *M. algicola* strains harbor notionally more ECRs (up to 11 ECRs [11]) than those of the new species (Table 1).

Since both novel strains KMM 9989^T^ and KMM 9879 contained one common plasmid (Figure 3C) and another strain-specific plasmid (Figure 3D,E), codon usage analysis was performed to determine whether the ECRs are chromids or megaplasmids. Both plasmids showed a G+C content and a codon usage very similar to those of the chromosome (Figure S3), allowing them to be classified as chromids [66]. In addition, they carried key genes responsible for pyruvate metabolism, biosynthesis of certain amino acids and cofactors, and rRNA methylation.

**Figure 4:**
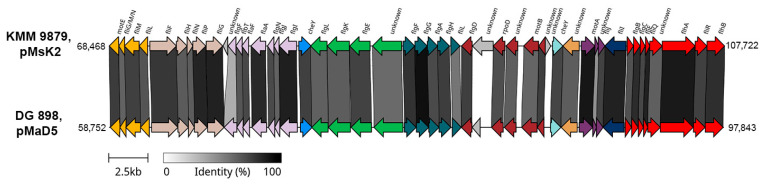
The comparison of *fla2*-type FGC organization for KMM 9879 and *M. algicola* DG898. The supposed operons are shown in colors. Gray bars between the ORFs represent their percentage of identity to each other, with darker color corresponding to higher percentage.

Remarkably, the genome of KMM 9879 contains a flagellar gene cluster (FGC), while the genome of KMM 9989^T^ does not. This finding is supported by the motility testing results, as KMM 9879 displayed an ability to move, and its micrograph showed the presence of bacterial cells with flagella (Section 3.4). This FGC was located on pMsK2 chromid (Figure 3E) and showed high sequence similarity to the FGC localized on pMaD5 chromid of *M. algicola* DG898 (from 50% for *flgF* up to 95% for *flgG* and *motA*) [62]. Their operon structure classified as a type 2 flagellar system (*fla2*-type) [64] was almost identical, including the presence of *rpoD* (RNA polymerase sigma-70 factor) and *cheY* (response regulator) genes in the same positions (Figure 4). This indicates a close relatedness of the *fla2*-type systems in KMM 9879 and DG898, despite their existence in different chromids.

However, this finding may represent a second case of strain-specific motility previously described for *Roseivivax marinus*; strain LMG 27156 (former *Roseivivax atlanticus*) was motile and had a complete *fla2*-type FGC, while the DSM 27511^T^ strain was non-motile and lacked the corresponding genetic apparatus [12]. Unfortunately, due to the incompleteness of the *Roseivivax marinus* LMG 27156 genome, we were unable to determine the localization of the *fla2*-type FGC. Thus, we propose that the motility detected only in KMM 9879 may be mediated by strain-specific plasmid. This unique case illustrates the remarkable depth of morphological changes that a single plasmid can induce in the *Rhodobacterales* bacteria.

**Figure 5:**
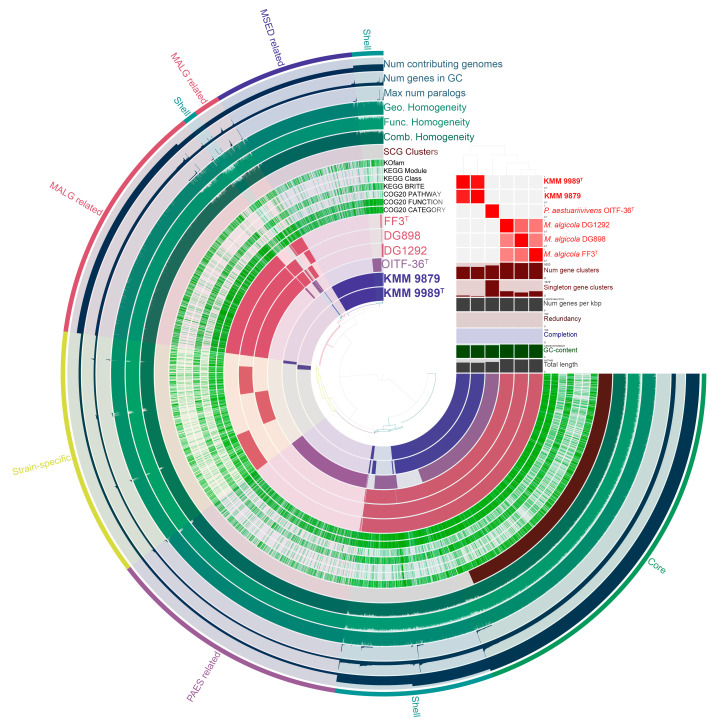
The pan-genome of six strains of the *Marinovum* clade generated with anvi’o [56]. Circle bars represent the presence/absence of gene clusters in each genome. Gene clusters are organized as core (green), shell (cyan), species-related (different colors), and strain-specific (yellow) ones using Euclidian distance and Ward ordination. *M. algicola* strains are colored in red and designated as MALG. KMM 9989^T^ and KMM 9879 strains are colored in violet and designated as MSED. *P. aestuarivivens* is colored in purple and designated as PAES. The heatmap in the upper right corner shows pairwise ANI values.

Assessment of the genus-associated features was performed through a pan-genome analysis (Figure 5) of the complete genome sequences of *Marinovum* strains using orthologous gene clustering and metabolic pathway reconstruction on the anvi’o platform [56]. It is worth noting that, based on the OGRIs values and phylogenomic position (Figure 2), *P. aestuariivivens* demonstrated a greater genetic relationship to the genus *Marinovum* than to the genus *Primorskyibacter*. Therefore, it was included in this analysis. The *Marinovum* pan-genome consisted of a total of 8435 gene clusters (GCs) with 27,989 gene calls. The core-genome included 2192 GCs covering 13,509 genes, of which 12,504 were single-copy genes (SCGs). The accessory shell and cloud were comprised of 1147 (5376 genes) and 5096 (9104 genes) clusters, respectively. The pan-genome unique (strain-specific) section encompassed 1403 GCs (1473 genes) of singletons. The largest and smallest number of singletons were detected in the *M. algicola* DG898 (480 genes) and KMM 9879 (50 genes). The annotated singletons in the novel genomes were mostly related to membrane transport (ABC-transporters, type II secretion system (T2SS)), carbohydrate metabolism, amino acid metabolism, transcription factors (LysR, LacI, and Lep/AsnC), type II toxin/antitoxin system (CcdA/CcdB), and enzymes (oxidoreductases, lyases, transferases, hydroxylases, hydrolases, chorismate mutase, isomerases, transglutaminases, dehydrogenases, and alkylhydroperoxidases).

Transport systems present in the novel strains were analyzed with MacSyFinder using the TXSSscan module [54]. Both new strains were found to possess T1SS, T2SS, and a tight adherence pilus system (TadPS). Interestingly, the T2SS operons were located dispersedly through the genomes. The first operon was located on the chromosome, the second was on the pMsK1 plasmid, and the third was present only the pMsK2 plasmid of KMM 9989^T^.

**Figure 6:**
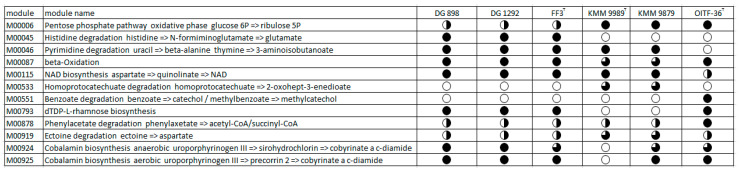
Discrimination of the *Marinovum* clade based on the completeness of predicted KEGG pathway modules. FF3^T^, DG898, and DG1292 are *M. algicola* strains. OITF-36^T^ is a *P. aestuariivivens* strain. The black-colored circle part corresponds to the pathway’s completeness, where a completely colored circle is 100%, and a white circle is 0%.

The metabolic pathways completeness was estimated using the anvi’o platform, then manually analyzed with Microsoft Excel and KAAS annotation data [58]. According to the obtained results (Figure 6), the pentose phosphate pathway was complete in the strains KMM 9989^T^, KMM 9879, and *P. aestuariivivens* OITF-36^T^, whereas it was partial in the *M. algicola* strains due to the absence of 6-phosphogluconate dehydrogenase [EC:1.1.1.44, 1.1.1.343], which catalyzes the conversion of D-glucose 6-phosphate to D-ribulose 5-phosphate. The histidine degradation pathway was completely absent in KMM 9989^T^, KMM 9879, and OITF-36^T^, while *M. algicola* strains had a complete one. The dTDP-L-rhamnose biosynthesis was not present only in the new strains. However, they possessed a pathway for ectoine degradation to aspartate, except for an N2-acetyl-L-2,4-diaminobutanoate deacetylase [EC:3.5.1.125]. There was also a remarkable difference between the new strains in the ability to synthesize cobalamin. The KMM 9879, like the *M. algicola* and *P. aestuariivivens* strains, had a complete pathway for aerobic cobalamin biosynthesis, while the KMM 9989^T^ did not. The fatty acid beta-oxidation pathway was complete in OITF-36^T^ and *M. algicola* strains, while the new strains lacked acetyl-CoA acyltransferase [EC:2.3.1.16] and butyryl-CoA dehydrogenase [EC:1.3.8.1]. Only the *P. aestuariivivens* OITF-36^T^ genome showed the capacity for benzoate degradation.

Despite the fact that *P. aestuariivivens* displayed higher genetic relatedness to the genus *Marinovum* than to the genus *Primorskyibacter* based on the OGRIs values (79.2% for ANI, 76.7% for AAI, and 18.5 for dDDH) and phylogenomic position (Figure 2), the predicted metabolic pathways do not support its placement in the genus *Marinovum*. Moreover, there are phenotypic distinctions of OITF-36^T^ from *Marinovum* strains, including halotolerant growth, proteolytic activity, phospholipid profile, and substrate assimilation spectrum [63]. Therefore, *P. aestuariivivens* OITF-36^T^ should be considered a representative of a new genus.

### 3.3. Comparative Functional Analysis

The functional analysis of the KMM 9989^T^ and KMM 9879 was performed using the Protologger server [67]. For the type strain KMM 9989^T^, among the 3819 coding sequences identified, 231 were predicted to be transporters, and 40 were considered secretion-associated. The genome was found to encode 939 unique enzymes. The KMM 9989^T^ was predicted to be capable of starch utilization, which, however, was not proven by biochemical testing (Section 3.4). On the other hand, oxidation of glucose through the Embden–Meyerhof pathway was identified, which was consistent with the results of biochemical analysis. The urease (EC:3.5.1.5) encoded cluster was identified. The strain was predicted to be capable of melibiose degradation, contrary to the biochemical testing results. According to the KEGG annotation, KMM 9989^T^ was able to produce hydrogen sulfide from thiosulfate through either Soe EC:1.8.5.6/EC:1.8.1.2 or Sox EC:2.8.1.1/EC:1.8.1.2 pathways. Interestingly, the type strain FF3^T^ of *M. algicola* had similar capabilities according to the genome analysis yet failed to display them during the biochemical testing. Similarly, FF3^T^, KMM 9989^T^, and KMM 9879 contained a path for sucrose oxidation (EC:3.2.1.20/EC:2.7.1.4/EC:5.3.1.9/EC:5.4.2.2) as well as a glycogen phosphorylase (EC:2.4.1.1), allowing for degradation of starch. However, FF3^T^ lacked the ability to oxidize D-sucrose. KMM 9989^T^ and KMM 9879 strains could not utilize starch during the biochemical tests.

The genomes of KMM 9989^T^ and KMM 9879 were also annotated with dbCAN server [51] to analyze their polysaccharide utilization potential. The new strains possessed an almost identical repertoire of carbohydrate-active enzymes (CAZymes). The KMM 9879 genome encoded 72 CAZyme genes, while the KMM 9989^T^ did 74. This discrepancy was due to the difference in two additional CAZymes predicted on pMsK2 plasmid of KMM 9989^T^. Among the identified CAZymes, 24 belonged to glycoside hydrolases (GH) classified into 17 families, 35 to glycosyltransferases of 13 families, two to polysaccharide lyases of two families, four to carbohydrate esterases (CE) of four families, and five to auxiliary activities of three families. The KMM 9989^T^ had additional GH109 and CE4.

### 3.4. Morphological, Physiological, and Biochemical Characteristics

**Figure 7:**
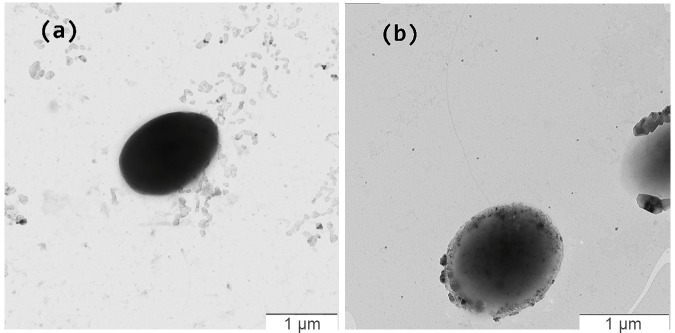
Transmission electron micrographs of strains KMM 9989^T^ (**a**) and KMM 9879 (**b**). Bar, 1 µm.

Strains KMM 9989^T^ and KMM 9879 were aerobic Gram-negative-stained whitish-pigmented spherical or ovoid-shaped bacterial cells, 1.2–1.5 μm in diameter, non-motile (strain KMM 9989^T^, Figure 7a) or motile with one to two polar or lateral flagella (strain KMM 9879) (Figure 7b and Figure S4).

In addition, the novel bacteria showed slight differences from each other in terms of temperature and salinity range for growth. Both strains were able to grow in the range of 0.5–4% NaCl and at 5–34 °C, while strain KMM 9879 could grow in 5% NaCl, and strain KMM 9989^T^ could grow at 35 °C. Neither novel strain could degrade gelatin, casein, starch, DNA, or Tweens 20, 40, or 80, reduce nitrate, or assimilate any of the carbon sources in the 32ID GN tests (Table 2). Physiological and biochemical characteristics of the novel bacteria are given in Table 1 and in the species description. The phenotypic characteristics that differentiate strains KMM 9989^T^ and KMM 9879 from related species are listed in Table 2.

### 3.5. Chemotaxonomic Characteristics

Strains KMM 9989^T^ and KMM 9879 contained the predominant ubiquinone Q-10 and the major fatty acid C_18:1_*ω7c* (>85%), and their fatty acid profiles obtained were similar to that of relative *M. algicola* KCTC 22095^T^ (Table 3). The polar lipids of strains KMM 9989^T^, KMM 9879, and *M. algicola* KCTC 22095T were alike and comprised phosphatidylcholine (PC), phosphatidylglycerol (PG), phosphatidylethanolamine (PE), diphosphatidylglycerol (DPG), and an unidentified aminolipid (AL), an unidentified phospholipid (PL2), and unidentified lipids (L2, L3). Strain KMM 9989^T^ did not contain PA and contained additionally an unidentified lipid L4 compared with two other relatives. In addition, polar lipids of strains KMM 9879 and *M. algicola* KCTC 22095^T^ included phosphatidic acid (PA), and unidentified phospholipids (PL3 and PL1, respectively) and *M. algicola* KCTC 22095^T^ contained an unidentified lipid (L1) (Figure S5). The DNA GC content of 61.3 and 61.4 mol% was calculated from the genome sequences of strains KMM 9989^T^ and KMM 9879, respectively.

These chemotaxonomic characteristics obtained for the novel bacteria, including ubiquinone Q-10; the predominance of C_18:1_*ω7c*; polar lipid components of PC, PG, PE, DPG, and AL; and the DNA GC content values, are consistent with those previously described for *M. algicola* [2,3], confirming their assignment to the genus *Marinovum*.

## 4. Conclusions

In summary, the phylogenetic and genetic distinctions found for the novel isolates KMM 9989^T^ and KMM 9879 were supported by phenotypic differences, including temperature and salinity ranges for their growth, enzyme activity, H_2_S production from thiosulfate, carbon source assimilation spectrum, and the ability of one of the strains to form lateral flagella (Table 2). Based on the combined phylogenetic and phylogenomic evidence and phenotypic traits, it is proposed to classify KMM 9989^T^ and KMM 9879 as a novel species, *Marinovum* *sedimenti* sp. nov.

**Description of** ***Marinovum sedimenti*** **sp. nov.**

*Marinovum sedimenti* (se.di.men’ti. L. gen. neut. n. *sedimenti*, of sediment, from where the type strain was isolated).

Aerobic, Gram-negative, catalase- and oxidase-positive, non-motile spherical or ovoid bacterial cells, 1.2–1.5 μm in diameter; some strains can be motile with one or two polar and/or lateral flagella. On MA 2216, formed whitish-pigmented colonies with regular edges of 3–4 mm in diameter. Growth occurs in 0.5–4% NaCl (*w*/*v*) and is optimal in 2–3% NaCl. Growth in 5% NaCl is strain-dependent, reaction of type strain is negative. The temperature range for growth was 5–35 °C, with an optimum of 28–30 °C. Growth at 35 °C is strain-dependent; reaction of type strain is positive. Weak growth was observed at 0.5% NaCl. The pH range for growth is pH 5.5–10.0, with an optimum of 6.5–7.5. Negative for hydrolysis of casein, gelatin, L-tyrosine, DNA, Tween 20, Tween 40, Tween 80, starch, nitrate reduction. Positive for H_2_S production from thiosulfate.

In the API 20E, positive for ONPG, oxidation of D-glucose, D-sucrose, and amygdalin and negative for arginine dihydrolase, lysine decarboxylase, ornithine decarboxylase, citrate utilization, H_2_S and urease production under anaerobic conditions, tryptophane deaminase, indole production, acetoin production, gelatin hydrolysis, and oxidation/fermentation of D-mannitol, inositol, D-sorbitol, L-rhamnose, D-melibiose, and L-arabinose.

In the ID32 GN tests, negative for the assimilation of sodium acetate, L-alanine, glycogen, L-serine, D-mannitol, D-glucose, L-histidine, L-proline, 3-hydroxybutyric acid, L-rhamnose, N-acetylglucosamine, D-ribose, inositol, D-sucrose, D-maltose, itaconic acid, suberic acid, sodium malonate, lactic acid, potassium 5-ketogluconate, 3-hydroxybenzoic acid, salicin, D-melibiose, L-fucose, D-sorbitol, L-arabinose, propionic acid, capric acid, trisodium citrate, potassium 2-ketogluconate, and 4-hydroxybenzoic acid.

API ZYM tests were positive for esterase C 4, esterase lipase C 8, and naphthol-AS-BI-phosphohydrolase; weakly positive for acid phosphatase; and negative for alkaline phosphatase, lipase C 14, valine arylamidase, cystine arylamidase, trypsin, α-chymotrypsin, α-galactosidase, β-galactosidase, β-glucuronidase, α-glucosidase, β-glucosidase, N-acetyl-β-glucosaminidase, α-mannosidase, and α-fucosidase. Leucine arylamidase activity is strain-dependent, and reaction of the type strain is positive.

The dominant respiratory quinone was ubiquinone Q-10. The major fatty acid was C_18:1_*ω7c*. The polar lipids consisted of phosphatidylcholine, phosphatidylglycerol, phosphatidylethanolamine, diphosphatidylglycerol, an unidentified aminolipid, one or two unidentified phospholipids, and three unidentified lipids. The presence of phosphatidic acid is strain-dependent, and the type strain did not contain PA.

The DNA GC content of 61.3–61.4% was calculated from the genome sequences.

The type strain of the species is strain KMM 9989^T^ (=KCTC 8835^T^), isolated from a bottom sediment collected from of the Okhotsk Sea (54.187933, 137.831566), Russia.

The DDBJ/GenBank accession number for the 16S rRNA gene sequence of strain KMM 9989^T^ is PP217372. The annotated complete genome of type strain KMM 9989^T^ comprising 4,040,543 bp is deposited in the NCBI GenBank database under the accession number JBSWBS000000000.

## Figures and Tables

**Table 1:** Genomic features of KMM 9989^T^, KMM 9879, and related strains from *Marinovum* and *Primorskyibacter* genera.

Feature	1	2	3	4	5	6
Assembly level	chromosome	chromosome	scaffold	complete	complete	contig
Genome size (bp)	4,040,543	3,969,839	5,396,756	5,477,406	5,252,390	4,292,765
Number of contigs	3	3	63	10	12	44
G+C Content (mol%)	61.3	61.4	65	65.5	65	61.5
N50 (Kb)	3613.5	3613.5	210.4	3887.0	3700.0	169.2
L50	1	1	8	1	1	7
Coverage	265x	341x	198x	3887x	32x	337x
Total genes	3869	3817	5267	5344	5066	4266
Protein coding genes	3708	3670	5131	5187	4939	4173
rRNAs (5S/16S/23S)	3/3/3	3/3/3	2/2/2	2/2/2	2/2/2	1/1/1
tRNA	49	49	45	46	45	46
checkM completeness (%)	100	100	99.69	99.69	99.36	100
checkM contamination (%)	0.29	0.29	0.31	0.31	0.59	1.89
WGS project/RefSeq	JBSWBS01	JBSWBT01	FNYY01	GCF_041429805.1-RS_2024_08_23	GCF_001046955.1	JAQIOZ01
Genome assembly/GenBank ID	ASM5405216v1	ASM5405220v1	IMG-taxon 2615840718	Genome assembly DG 1292	ASM104695v1	ASM2802367v1

Strains: **1**, KMM 9989^T^; **2**, KMM 9879; **3**, *M. algicola* FF3^T^; **4**, *M. algicola* DG 1292; **5**, *M. algicola* DG 898 **6**, *P. aestuariivivens* OITF-36^T^.

**Table 2:** Differential characteristics of strains KMM 9989^T^ and KMM 9879 and *M. algicola* KCTC 22095^T^.

Characteristic	1	2	3
DNA GC content (%) *	61.3	61.4	65
Motility	−	+	+
Maximal growth temperature (°C)	35	34	37
Maximal NaCl concentration (%)	4	5	8
Nitrate reduction	−	−	+
H_2_S production	+	+	−
Starch hydrolysis	−	−	(+)
API 20E			
Oxidation of:			
D-Glucose	+	+	−
D-Sucrose	+	+	−
Amygdalin	+	+	−
Enzyme activity (API ZYM):			
Leucine arylamidase	+	−	−
Acid phosphatase	(+)	(+)	−

Strains: 1, KMM 9989^T^; 2, KMM 9879; 3, *M. algicola* KCTC 22095^T^ (data were obtained from present study unless otherwise indicated). +, Positive; −, negative; (+), weak reaction r. All strains were positive for catalase and oxidase and negative for growth at 4 °C and hydrolysis of gelatin, casein, tyrosine, DNA, and Tween-80; in the API ZYM, tests were positive for esterase C4, esterase lipase C8, and naphthol-AS-BI-phosphohydrolase and negative for alkaline phosphatase, lipase C14, valine arylamidase, cystine arylamidase, trypsin, α-chymotrypsin, α-galactosidase, β-galactosidase, β-glucuronidase, α-glucosidase, β-glucosidase, N-acetyl-β-glucosaminidase, α-mannosidase, and α-fucosidase. * The DNA GC contents of the strains KMM 9989^T^, KMM 9879, and KCTC 22095^T^ were derived from their genome assembly data (Table 1).

**Table 3:** Fatty acid composition (%) of strains KMM 9989^T^, KMM 9879, and *M. algicola* KCTC 22095^T^.

Fatty Acid	1	2	3
C_10:1_*ω8*	0.08	1.44	0.92
C_12:0_ 3-OH	0.26	0.44	1.02
C_16:0_	3.80	4.58	3.36
C_18:1_*ω7c*	89.11	86.69	84.69
C_18:0_	1.76	2.01	2.83
11-Methyl C_18:1_*ω7c*	1.53	1.09	2.98

Strains: 1, KMM 9989^T^; 2, KMM 9879; 3, *M. algicola* KCTC 22095^T^ (data from the present study). Fatty acids representing <1% in all strains tested are not shown.

## Data Availability

The type strain of the species is strain KMM 9989^T^ (=KCTC 8835^T^), isolated from a bottom sediment collected from of the Okhotsk Sea, Russia. The DDBJ/GenBank accession numbers for the 16S rRNA gene sequences of strains KMM 9989^T^ and KMM 9879 are PP217372 and PP217369, respectively. The DDBJ/GenBank accession numbers for the genome sequences of strains KMM 9989^T^ and KMM 9879 are JBSWBS000000000 and JBSWBT000000000, respectively.

## Supplementary Materials

The following supporting information can be downloaded at https://www.mdpi.com/article/10.3390/microorganisms14010125/s1, Table S1: List of bacterial genomes used for ML phylogenomic tree (Figure 1); Figure S1: A whole-proteome-based tree of KMM 9989^T^ and related strains generated by TYGS server; Figure S2: ML tree based on concatenated sequences of 400 proteins showing phylogenetic affiliation of CGMCC 1.12644^T^ and CGMCC 1.12664^T^ (shown in bold) to *Primorskyibacter* and *Pseudooceanicola* genera, respectively. Bootstrap values are based on 100 replicates. Bar, 0.20 substitutions per amino acid position; Figure S3: Codon usage for KMM 9989^T^ and KMM 9879 replicons calculated using an online calculator available at https://www.bioinformatics.org/sms2/codon_usage.html, accessed on 18 December 2025; Figure S4: Transmission electron micrographs of strain KMM 9879. Bar, 1 µm. Figure S5: Two-dimensional thin-layer chromatograms of polar lipids of strains: (a–c) *M. algicola* KCTC 22095^T^; (d–f) KMM 9989^T^; (g–i) KMM 9879. (a,d,g), non-specific detection of lipids prepared with 10% H_2_SO_4_ in methanol; (b,e,h), stained with ninhydrin; (c,f,i), stained with a molybdate reagent. Abbreviations: PC, phosphatidylcholine; PG, phosphatidylglycerol; PE, phosphatidylethanolamine; DPG, diphosphatidylglycerol; PA, phosphatidic acid; AL, an unidentified aminolipid; PL, PL1, PL2, unidentified phospholipids; L1, L2, L3, L4, unidentified lipids.
