# Supplementary material for: Genomic Insights into Marinovum sedimenti sp. nov., Isolated from Okhotsk Sea Bottom Sediments, Suggest Plasmid-Mediated Strain-Specific Motility
Source: Microorganisms. 2026 Jan 7;14(1):125. doi: 10.3390/microorganisms14010125 (PMC12843797; doi:10.3390/microorganisms14010125)
Supplement: Supplementary file 1 [file microorganisms-14-00125-s001.zip › microorganisms-4056684-supplementary.pdf]

Genomic Insights into *Marinovum sedimenti* sp. nov., Isolated from Okhotsk Sea Bottom Sediments, Suggest Plasmid-Mediated Strain-Specific Motility

Lyudmila Romanenko [1,*], Viacheslav Eremeev [1], Evgeniya Bystritskaya [1], Peter Velansky [2], Valeriya Kurilenko [1], and Marina Isaeva [1,*]

[1] G.B. Elyakov Pacific Institute of Bioorganic Chemistry, Far Eastern Branch, Russian Academy of Sciences, Prospect 100 Let Vladivostoku, 159, Vladivostok 690022, Russia; lro@piboc.dvo.ru (L.R.); wieremeew@gmail.com (V.E.); ep.bystritskaya@yandex.ru (E.B.); valerie@piboc.dvo.ru (V.K.); issaeva@gmail.com (M.I.)

[2] A.V. Zhirmunsky National Scientific Center of Marine Biology, Far Eastern Branch, Russian Academy of Sciences, Palchevskogo Street 17, Vladivostok 690041, Russia; velansky.pv@gmail.com (P.V.)

* Correspondence: lro@piboc.dvo.ru (L.R.); issaeva@gmail.com (M.I.); Tel.: +7-423-231-1168 (L.R.)

Supplementary Materials

Table S1. List of bacterial genomes used for ML phylogenomic tree (Figure 1).

Species name	Genome ID	Size, Mb	Completeness, %
Ruegeria atlantica CECT 4292^{T}	GCF_001458195.1	4.8	96.8
Pseudophaeobacter arcticus DSM 23566^{T}	GCF_000473205.1	5.0	99.56
Phaeobacter gallaeciensis DSM 26640^{T}	GCF_000511385.1	4.5	98.28
Pseudosulfitobacter pseudonitzschiae DSM 26824^{T}	GCF_900129395.1	5.0	99.34
Marinovum algicola FF3^{T}	GCF_900109145.1	5.4	99.69
Primorskyibacter aestuariivivens OITF-36^{T}	GCF_028023675.1	4.3	100.0
Primorskyibacter marinus PX7^{T}	GCF_003313245.1	4.1	99.54
Primorskyibacter sedentarius DSM 104836^{T}	GCF_004342065.1	5.1	100.0
Marivita cryptomonadis CL-SK44^{T}	GCF_002115725.1	4.6	99.69
Alloyangia pacifica DSM 26894^{T}	GCF_900116195.1	6.1	99.39
Pseudoprimorskyibacter insulae CECT 8871^{T}	GCF_900302505.1	4.0	99.12
Puniceibacterium antarcticum SM1211^{T}	GCF_002760615.1	5.5	99.49
Roseivivax halodurans JCM 10272^{T}	GCF_000521785.1	4.5	98.93
Thalassococcus halodurans DSM 26915^{T}	GCF_900108225.1	4.0	99.69
Mameliella alba DSM 26384^{T}	GCF_003051025.1	5.3	99.98
Antarctobacter heliothermus DSM 11445^{T}	GCF_900188425.1	5.2	100.0
Maliponia aquimaris CECT 8898^{T}	GCF_900184945.1	5.3	99.71
Pseudoponticoccus marisrubri SJ5A-1^{T}	GCF_001482405.1	4.6	99.67
Pelagimonas varians DSM 23678^{T}	GCF_003208435.1	4.9	99.54
Tropicibacter naphthalenivorans CECT 7648^{T}	GCF_001458375.1	4.5	99.39
Thetidibacter halocola KMU-90^{T}	GCF_018224955.1	4.8	99.69
Poseidonocella pacifica DS M 2931 e^{T}	GCF_900111875.1	3.6	100
Roseovarius tolerans DSM 11457^{T}	GCF_900109855.1	3.8	93.22
Seohaeicola saemankumensis CCUG 55328^{T}	GCF_042678485.1	3.9	99.24
Primorskyibacter flagellatus CGMCC 1.12664^{T}	GCF_014638275.1	4.6	99.69
Caulobacter vibrioides DSM 9893^{T}	GCF_002858865.1	4.0	93.39

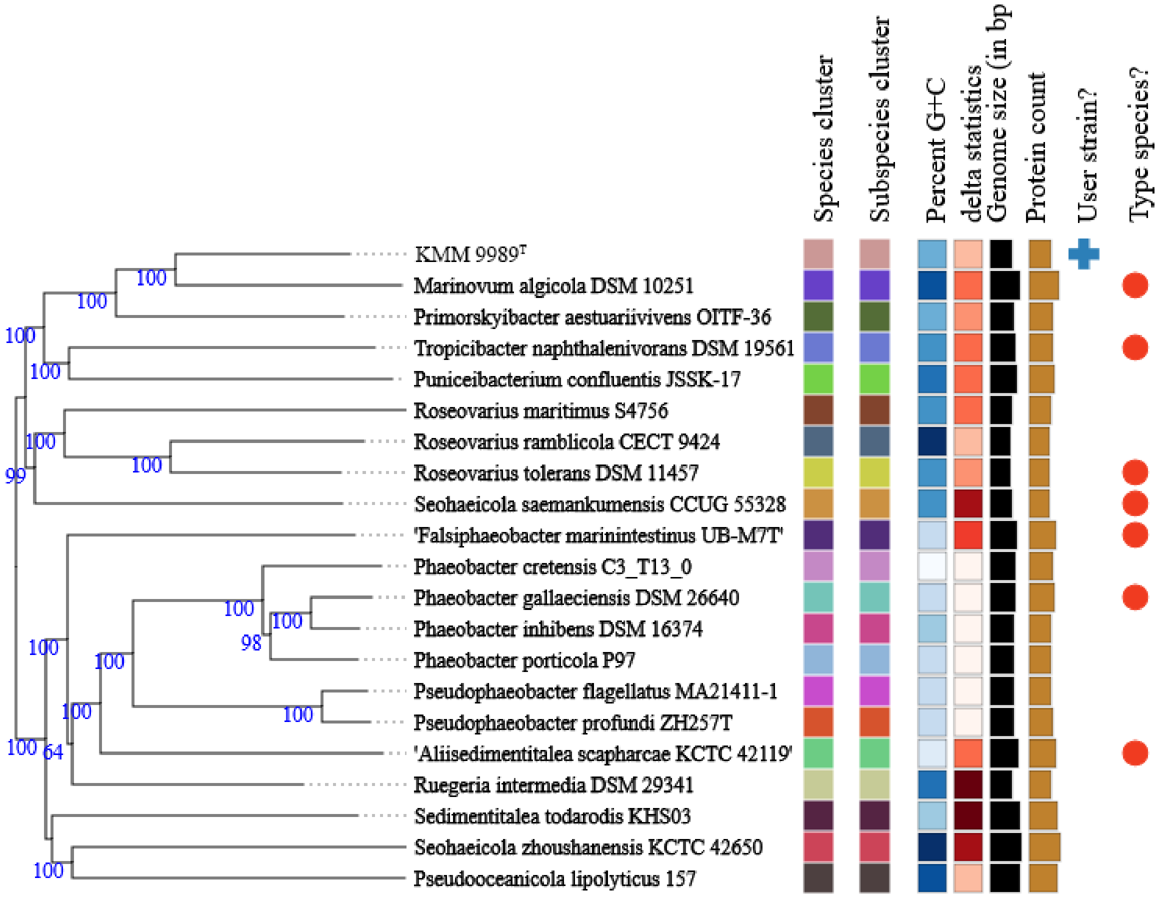

Figure S1. A whole-proteome-based tree of KMM 9989^T and related strains generated by TYGS server.

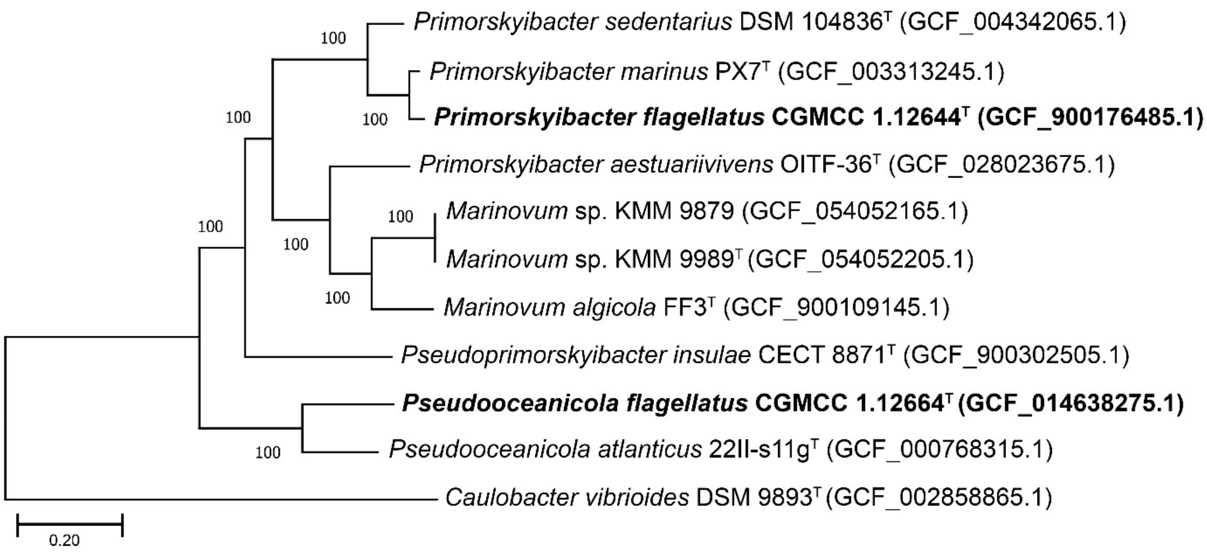

Figure S2. ML tree based on concatenated sequences of 400 proteins showing phylogenetic affiliation of CGMCC 1.12644^T and CGMCC 1.12664^T (shown in bold) to *Primorskyibacter* and *Pseudooceanicola* genera, respectively. Bootstrap values are based on 100 replicates. Bar, 0.20 substitutions per amino acid position.

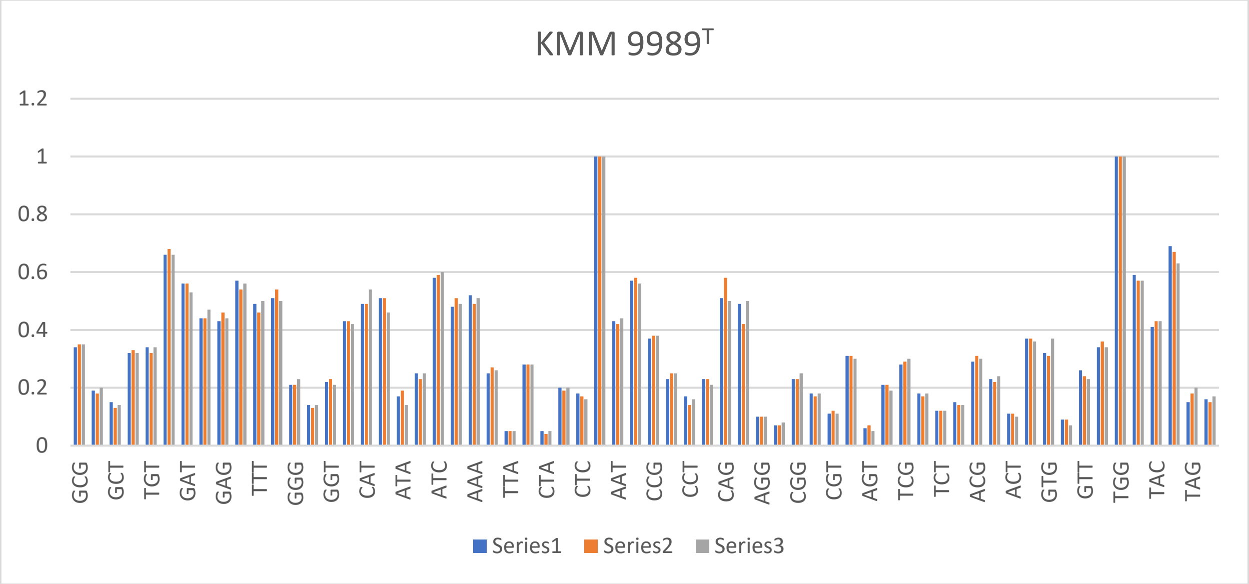

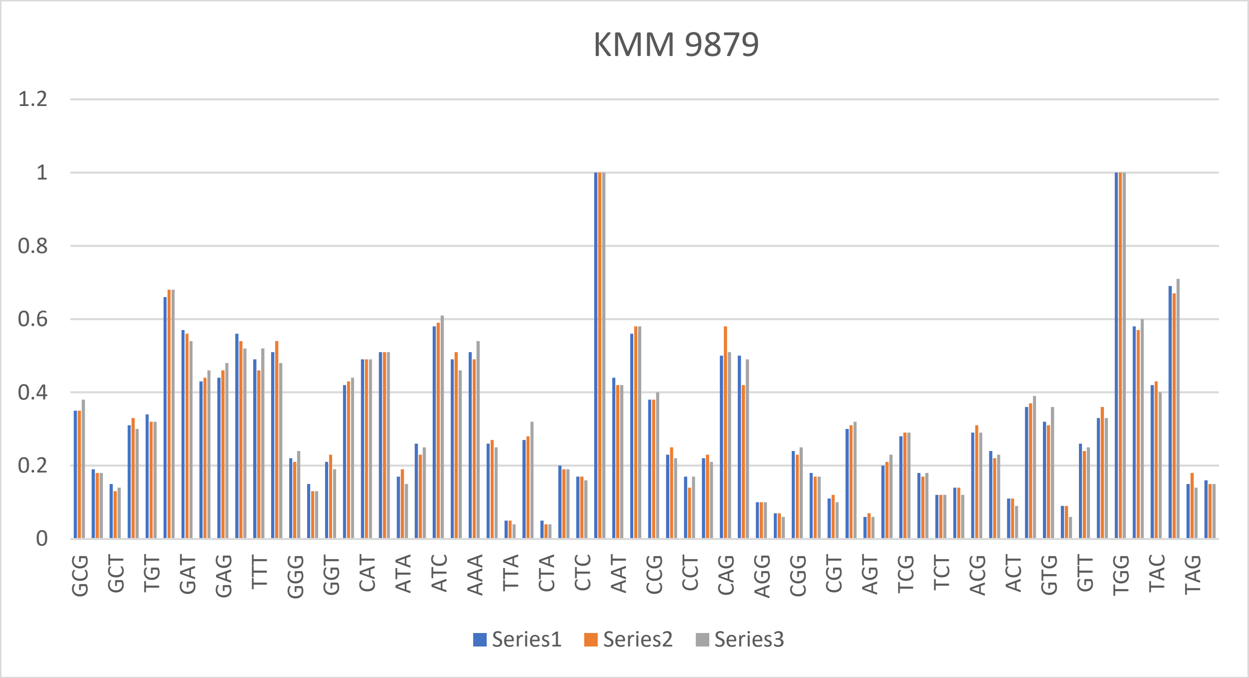

Figure S3. Codon usage for KMM 9989^T and KMM 9879 replicons calculated using an online calculator available at https://www.bioinformatics.org/sms2/codon_usage.html

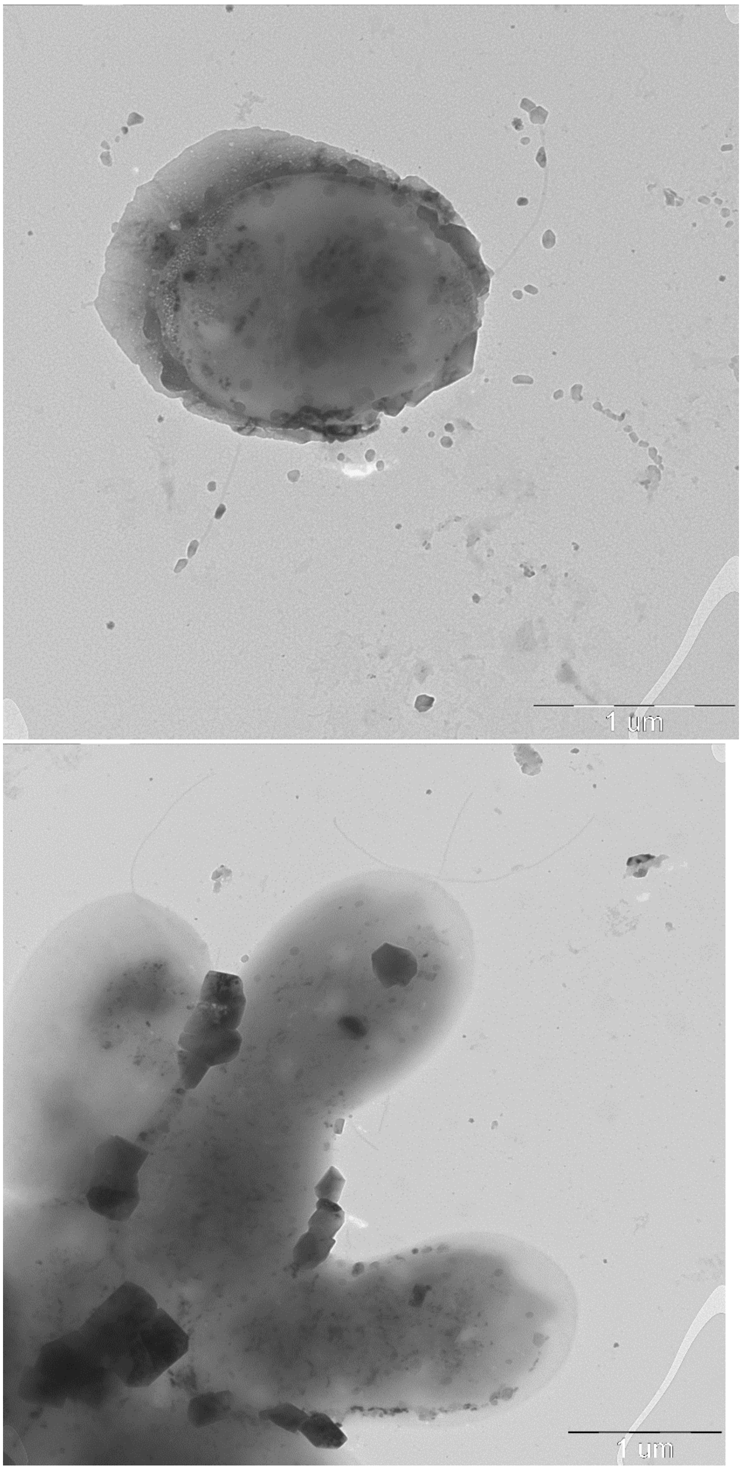

Figure S4. Transmission electron micrographs of strain KMM 9879. Bar, 1 µm.

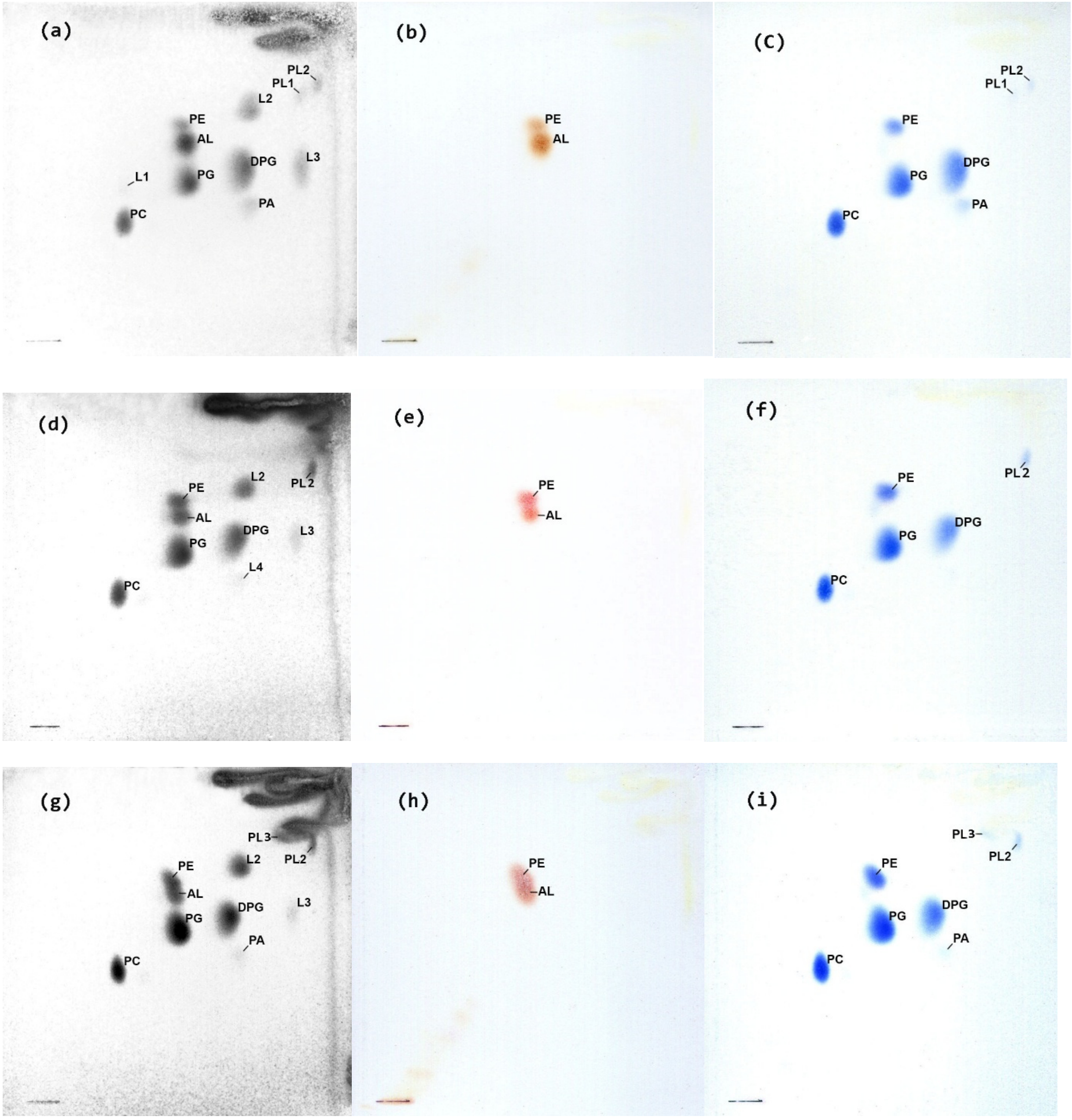

Figure S5. Two-dimensional thin-layer chromatograms of polar lipids of strains: (a, b, c) *M. algicola* KCTC 22095^T; (d, e, f) KMM 9989^T; (g, h, i) KMM 9879. (a, d, g), non-specific detection of lipids prepared with 10% H_2SO_4 in methanol; (b, e, h), stained with ninhydrin; (c, f, i), stained with a molybdate reagent. Abbreviations: PC, phosphatidylcholine; PG, phosphatidylglycerol; PE, phosphatidylethanolamine; DPG, diphosphatidylglycerol; PA, phosphatidic acid; AL, an unidentified aminolipid; PL, PL1, PL2, unidentified phospholipids; L1, L2, L3, L4, unidentified lipids.
